# Comparative study of ^64^Cu/NOTA-[D-Tyr^6^,βAla^11^,Thi^13^,Nle^14^]BBN(6-14) monomer and dimers for prostate cancer PET imaging

**DOI:** 10.1186/2191-219X-2-8

**Published:** 2012-02-14

**Authors:** Patrick Fournier, Véronique Dumulon-Perreault, Samia Ait-Mohand, Réjean Langlois, François Bénard, Roger Lecomte, Brigitte Guérin

**Affiliations:** 1 Centre d'imagerie moléculaire de Sherbrooke (CIMS), Department of Nuclear Medicine and Radiobiology, Université de Sherbrooke, 3001, 12th North Avenue, Sherbrooke, Quebec, J1H 5N4, Canada; 2 BC Cancer Agency Research Centre, 675 West 10th Avenue, Vancouver, British Columbia, V5Z 1L3, Canada

**Keywords:** Bombesin, homo-dimer, ^64^Cu, PET imaging, gastrin-releasing peptide receptors, PC3 tumor.

## Abstract

**Background:**

Gastrin-releasing peptide receptors [GRPR] are highly over-expressed in multiple cancers and have been studied as a diagnostic target. Multimeric gastrin-releasing peptides are expected to have enhanced tumor uptake and affinity for GRPR. In this study, a ^64^Cu-labeled 1,4,7-triazacyclononane-1,4,7-triacetic acid [NOTA]-monomer and two NOTA-dimers of [D-Tyr^6^,βAla^11^, Thi^13^, Nle^14^]bombesin(6-14) ] [BBN(6-14)] were compared.

**Methods:**

Monomeric and dimeric peptides were synthesized on solid phase support and radiolabeled with ^64^Cu. NOTA-dimer 1 consists of asymmetrically linked BBN(6-14), while NOTA-dimer 2 has similar spacer between the two BBN(6-14) ligands and the chelator. *In vitro *GRPR-binding affinities were determined with competitive binding assays on PC3 human prostate cancer cells. *In vivo *stability and biodistribution of radiolabeled compounds were assessed in Balb/c mice. Cellular uptake and efflux were measured with radiolabeled NOTA-monomer and NOTA-dimer 2 on PC3 cells for up to 4 h. *In vivo *biodistribution kinetics were measured in PC3 tumor-bearing Balb/c nude mice by μ-positron emission tomography [μPET] imaging and confirmed by dissection and counting.

**Results:**

NOTA-monomer, NOTA-dimers 1 and 2 were prepared with purity of 99%. The inhibition constants of the three BBN peptides were comparable and in the low nanomolar range. All ^64^Cu-labeled peptides were stable up to 24 h in mouse plasma and 1 h *in vivo*. ^64^Cu/NOTA-dimer 2 featuring a longer spacer between the two BBN(6-14) ligands is a more potent GRPR-targeting probe than ^64^Cu/NOTA-dimer 1. PC3 tumor uptake profiles are slightly different for ^64^Cu/NOTA-monomer and ^64^Cu/NOTA-dimer 2; the monomeric BBN-peptide tracer exhibited higher tumor uptake during the first 0.5 h and a fast renal clearance resulting in higher tumor-to-muscle ratio when compared to ^64^Cu/NOTA-dimer 2. The latter exhibited higher tumor-to-blood ratio and was retained longer at the tumor site when compared to ^64^Cu/NOTA-monomer. Lower ratios of tumor-to-blood and tumor-to-muscle in blocking experiments showed GRPR-dependant tumor uptake for both tracers.

**Conclusion:**

Both ^64^Cu/NOTA-monomer and ^64^Cu/NOTA-dimer 2 are suitable for detecting GRPR-positive prostate cancer *in vivo *by PET. Tumor retention was improved *in vivo *with ^64^Cu/NOTA-dimer 2 by applying polyvalency effect and/or statistical rebinding.

## Background

Prostate cancer is the most frequently diagnosed cancer and the second leading cause of cancer-related deaths for males in the USA. One promising approach in prostate cancer diagnosis is the utilization of target-specific radiolabeled peptides for positron emission tomography [PET] imaging. Previous researches have shown that bombesin [BBN] analogs can be used to target gastrin-releasing peptide receptors [GRPR] with high affinity and selectivity. Gastrin-releasing peptide [GRP] is a 27-amino acid peptide that displays a wide range of physiological effects, including gastric and pancreatic secretions, nervous system stimulation, smooth muscle contraction, blood pressure and the regulation of cell growth in some malignant cell lines [1,2]. The presence of GRPR has been documented in small cell lung cancers [3], prostate cancers [4,5], breast cancers [6-8] and others [9]. In prostate cancer, the GRPR expression has been tied to neoplastic transformation [10], cell migration [11,12], proliferation [10,13] and invasion capacity [14-16]. GRPR is overexpressed on 84% of all human prostate cancers according to a study by Markwalder and Reubi [5]. These receptors represent an interesting molecular target for radiolabeled BBN analogs as diagnostic or radiotherapeutic applications for prostate cancer. BBN, a 14-amino acid-potent GRPR agonist found in the skin of the fire-bellied toad *Bombina bombina*, was first described by Anastasi et al. [17]. BBN is involved in regulating exocrine secretion, smooth muscle contraction and gastrointestinal hormone release [18], and it is widely expressed in the central nervous system [19]. [D-Tyr^6^,βAla^11^, Thi^13^, Nle^14^]BBN(6-14) [BBN(6-14)] is a potent modified GRPR agonist peptide that binds to GRPR with high affinity [20]. Various BBN analogs have been labeled with radiometals and used for PET imaging of GRPR-positive tumors. Schuhmacher et al. labeled a 1,4,7,10-tetraazacyclododecane-*N, N', N'', N'''*-tretraacetic acid [DOTA]-PEG [polyethylene glycol]_2_-BBN(6-14) with ^68^Ga [21] for PET imaging, while Chen et al. used DOTA-Lys^3^-bombesin with ^64^Cu [22]. Smith et al. successfully labeled modified BBN(7-14) analogs with ^64^Cu for potential use in diagnostic imaging using 1,4,7-triazacyclononane-1,4,7-triacetic acid [NOTA] or NO2A as chelating agents and obtained stable compounds [23,24].

To improve peptide-binding affinity, a multivalency approach has been introduced [25]. Traditionally, this approach involves the use of peptide homodimers or homomultimers in which peptide ligands of the same type are constructed with suitable linkers. The key for bivalency, binding to two receptors at the same time, is the distance between the two peptide motifs. The ability of a dimer peptide to achieve bivalency depends also on the receptor density [25]. If the receptor density is very high, the distance between two neighboring receptor sites will be short, which makes it easier for the dimer peptide to achieve the bivalency. While GRPR density on PC3 tumor cells is widely documented *in vitro *and *in vivo*, its expression is heterogeneous making it difficult to establish an average distance between the receptors [4]. Even if the distance between the two peptide motifs is not optimal, the local BBN peptide levels may still be high in the vicinity of GRPR sites once the first BBN ligand is bound. The detachment of the dual action ligand from the receptor is more likely to be followed by re-attachment if there are GRPR binding copies close to it, resulting in higher receptor affinity for homodimers and better tumor uptake with longer tumor retention [26]. Potential benefits of multimeric targeting peptides are accepted, but many questions concerning the mechanisms are still to be answered. A few studies on BBN-based homodimers have been reported with varying results. Carrithers and Lerner observed modest improvement in affinity for GRPR with their homodimer [27], while Gawlak et al. noted no difference in affinity between their monomer and homodimer [28]. Abiraj et al. observed higher cellular uptake and retention of their homodimers radiolabeled with ^177^Lu on GRPR-over-expressing PC3 cells [29].

**Figure 1:**
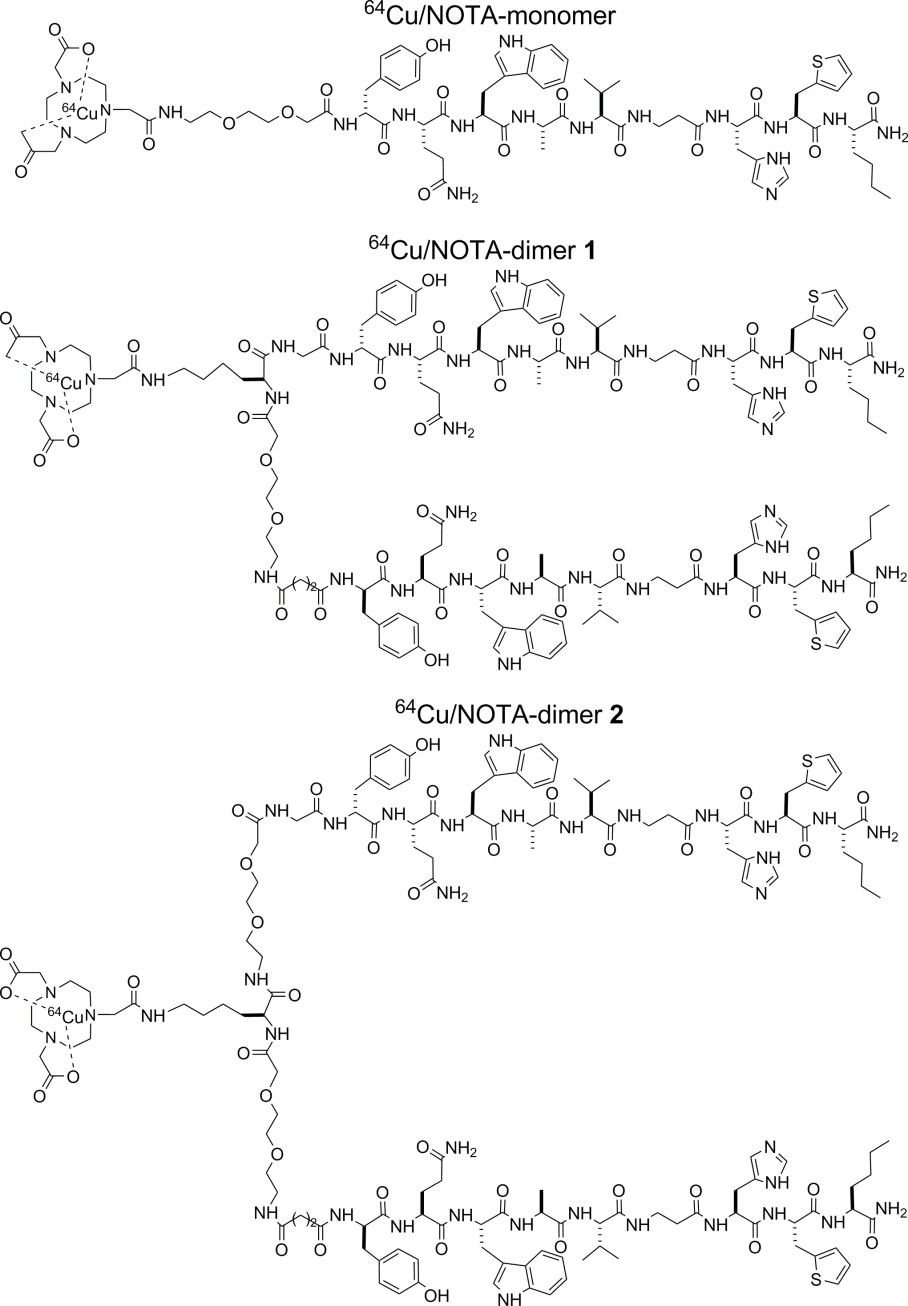
**Amino-acid sequences of NOTA-bombesin monomer and dimers with ^64^Cu**.

**Figure 2:**
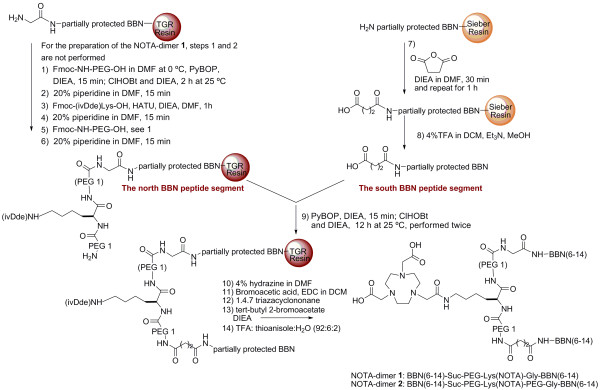
**Synthesis scheme for NOTA BBN-based dimers**.

^64^Cu has mean positron energy similar to that of ^18^F and a half-life of 12.7 h permitting PET evaluation of slow bio-chemical pathways, such as protein and peptide interactions with cellular targets [30]. Our laboratory has reported the synthesis and the characterization of DOTA and NOTA-BBN derivatives and showed that the NOTA-BBN(6-14) had an inhibition constant [K_i_] value slightly lower than that of the analog DOTA-BBN(6-14) [31]. NOTA has been radiolabeled efficiently with ^64^Cu and shown to have higher resistance to transmetallation reactions *in vivo *as compared to DOTA [23,35]. In the present study, we studied the GRPR affinity, the cellular uptake and efflux, the *in vivo *stability and the biodistribution of radiolabeled NOTA-BBN monomer and NOTA-BBN homodimers 1 and 2 which differed by the spacer length between the two peptide ligands (Figure 1). Our design is based on the only example available of radiolabeled BBN-based homodimer from Abiraj et al. [29]. They used lysine side chain or a 6-aminohexanoic acid spacer for their homodimer and obtained promising *in vitro *results. We used one and two PEG spacers between the binding sequences to fine-tune biological properties of our homodimers. In addition, we reported a convenient synthetic approach for the preparation of two NOTA-BBN homodimers and their labeling with ^64^Cu (Figure 2). μPET imaging on PC3 human prostate carcinomas xenografted in Balb/c nude mice was also performed to compare the diagnostic properties of ^64^Cu/NOTA-BBN homodimers homodimers to those of the ^64^Cu/NOTA-BBN monomer.

## Methods

### Materials

All chemicals and solvents (reagent grade) were used as supplied from the vendors cited below without further purification, unless otherwise noted. NovaSyn^® ^TGR resin and Sieber amide resin were obtained from EDM/NovaBiochem(Gibbstown, NJ, USA). Fmoc-protected amino acids and benzotriazol-1-yl-oxytripyrrolidinophosphonium hexafluorophosphate [PyBOP] were obtained from EMD NovaBiochem^® ^(Gibbstown, NJ, USA) or Chem-Impex International Inc. (Wood Dale, IL, USA). 1,4,7-Triazacyclononane was obtained from TCI America (Portland, OR, USA). 2-(1H-7-Azabenzotriazol-1-yl)-1,1,3,3-tetramethyluronium hexafluorophosphate [HATU] was purchased from Chem-Impex International Inc., and 6-Chloro-1-hydroxy-1H-benzotriazole [ClHOBT] was purchased from ChemPep (Wellington, FL, USA) and Matrix Innovation (Quebec, QC, CA). 4-(2-Hydroxyethyl)-1-piperazineethanesulfonic acid [HEPES], amphothericin B, Ham's F-12, phosphate-buffered saline [PBS], trypsin, penicillin, streptomycin and fetal bovine serum were purchased from Wisent (St-Bruno, Quebec, Canada). *N, N*-Diisopropylethylamine [DIEA], thioanisole were obtained from Aldrich Chemical Company, Inc. (Milwaukee, WI, USA). Bovine serum albumin [BSA] and bombesin were purchased from Sigma-Aldrich Company (Saint-Louis, MO, USA). Acetonitrile [MeCN], dichloromethane [DCM], *N, N*-dimetylformamide [DMF] and isopropyl alcohol were obtained from Fisher Scientific (Ottawa, Ontario, Canada). ^125^I-bombesin was purchased from Perkin Elmer Life Science Products (Boston, MA, USA). Finally, T47D human breast cancer and PC3 cell lines were obtained from American Type Culture Collection (Manassas, VA, USA). DMF was dried over 4 Å molecular sieves at least 1 week to remove trace amount of amine present in the solvent and filtered before its use.

### Peptide Synthesis

We recently reported the synthesis, the characterization and the biological activity of NOTA-BBN(6-14) peptide [31] . The general procedure for the preparation of NOTA-BBN(6-14) dimers on solid support is summarized in Figure 2. The south BBN peptide segment was synthesized on amide Sieber resin by a continuous flow method on a Pioneer™ Peptide Synthesis System (PerSeptive Biosystems; Framingham, MA, USA) using the Fmoc strategy. A two-fold excess of Fmoc-protected amino acid over available resin substitution sites was used for coupling in amine-free DMF. Fmoc-protected amino acids were activated for coupling with an equi-molar amount of HATU and two equivalents of DIEA. Fmoc deprotection was performed in 20% piperidine in DMF and monitored through absorbance at 364 nm. The resin was washed three times with DMF, MeOH, DMF, MeOH and DCM, subsequently. The partially protected peptide-resin was swelled in 2 mL of DMF and, then, treated with 3 mL of DMF containing succinic anhydride (10 equivalents) and DIEA (10 equivalents). This coupling procedure was performed twice (30 min and 1 h). The resin was washed as described above, and the desired peptide was cleaved from the support by treatment with a cocktail of 4% trifluoroacetic acid [TFA] in10 mL DCM at room temperature under mechanical agitation for 3 min. The solution was filtered into a flask containing 5% Et_3_N in MeOH. The cleavage step was repeated 10 times. Combined filtrates containing the partially protected peptide were evaporated under reduced pressure to 5% of the volume. Cold water (40 mL) was added to the residue, and the mixture was cooled with ice to aid the precipitation of the product. The precipitated peptides were centrifuged at 1,200 rpm for 15 min. The water solution was decanted, and the white solid was dried under vacuum. Purity of the crude peptide was verified by high performance liquid chromatography [HPLC] and, its identity was confirmed by API 3000 LC/MS/MS (Applied Biosystems/MDS SCIEX, Concord, Ontario Canada).

The north BBN peptide segment was synthesized on NovaSyn^® ^TGR resin and the automated system following the procedure described above. After completion of the BBN fragment, the Fmoc-NH-(PEG)_1_-CO_2_H and Fmoc (ivDde)Lys-OH were coupled manually to the peptide on resin. The Fmoc-NH-(PEG)_1_-CO_2_H (2.5 equivalents) was dissolved in 2 mL of DMF at 0°C, DIEA (2.5 equivalents) and PyBOP (2.5 equivalents) were added to the cold solution. After 15 min of stirring, the mixture was added to the partially protected peptide-resin pre-swelled with DCM and mixed with ClHOBt (2.5 equivalents) and DIEA (2.5 equivalents), while mechanical agitation was maintained for 2 h at room temperature. The resin was washed three times with DMF, MeOH, DMF, MeOH and DCM, subsequently. Fmoc deprotection was performed in 20% piperidine in DMF during 15 min, and the resin was washed as described above. For the preparation of the NOTA-dimer 1, steps 1 and 2 of Figure 2 were not performed. The Fmoc-Lys(ivDde)-OH was dissolved in 2 mL of DMF at 0°C, and HATU (2.5 equivalents) was added to the cold solution. The mixture was added to the partially protected peptide-resin pre-swelled with DCM and DIEA (2.5 equivalents) and, then, mechanically stirred for 1 h. The resin, the Fmoc group and the last Fmoc-NH-(PEG)_1_-CO_2_H were respectively washed, deprotected and coupled as described above. The resulting *N*-terminal Fmoc was deprotected in 20% piperidine in DMF for 15 min, and the resin was washed as described above. The coupling and the Fmoc deprotection steps were followed by a Kaiser's test on resin; the reaction between resin and ninhydrin was followed colorimetrically whereby free primary amines after Fmoc deprotection were detected as blue beads, and their absence as yellow beads. The resin was washed as described above.

### Coupling of the north BBN segment to the south peptide

The solution of HO-Suc-partially protected-BBN(6-14) was activated with PyBOP (1.5 equivalents), ClHOBt (1.5 equivalents) and DIEA (3 equivalents) in DMF:NMP (1:1 *v/v*). The pre-activation mixture was stirred for 15 min and, then, added to the NH_2_-PEG-Lys(ivDde)-(PEG)-Gly-BBN-peptide on TGR resin pre-swelled in DMF (2 mL). The reaction was allowed to proceed for 12 h at room temperature under mechanical agitation. The coupling was performed twice with another equivalent of the HO-Suc-partially protected-BBN(6-14). After the north BBN segment coupling, the NOTA group was built on solid phase as described previously by our group [31]. The resin was washed as described above, and the peptide was deprotected and cleaved from the support by treatment with a cocktail of TFA/H_2_O/thioanisole (92:2:6, *v/v/v*) for 4 h at room temperature under mechanical agitation to yield the desired peptide. The resin was removed by filtration and washed with TFA. Combined filtrates were added dropwise to cold diethyl ether. For each 1 mL of TFA solution, 10 mL of diethyl ether was used. The precipitated peptides were centrifuged at 1,200 rpm for 15 min. The ether solution was decanted, and the white solid was dissolved in water, frozen and lyophilized. The crude peptide was purified by flash chromatography on a Biotage SP4 system (Biotage, Charlotte, NC, USA) equipped with a C_18 _column. Purity of the peptides was verified by HPLC, and their identity was confirmed by API 3000 LC/MS/MS (Applied Biosystems/MDS SCIEX) and MALDI. Analytical HPLC was performed on an Agilent 1200 system (Agilent Technologies, Mississauga, Ontario, L5N 5M4, Canada) equipped with a Zorbax Eclipse XDB C18 reversed-phase column (4.6 × 250 mm, 5 μ) and Agilent 1200 series diode array UV-Vis detector (Agilent Technologies) using a linear gradient of 0% to 100% acetonitrile in water with 0.1% TFA over 30 min at a flow rate of 1 mL/min. Following these methods, NOTA-PEG-BBN(6-14) (denoted as NOTA-monomer), BBN(6-14)-Suc-PEG-Lys(NOTA)-Gly-BBN(6-14) (denoted as NOTA-dimer 1) and BBN(6-14)-Suc-PEG-Lys(NOTA)-PEG-Gly-BBN(6-14) (denoted as NOTA-dimer 2) were prepared.

### Cell culture

The human prostate cancer PC3 cell line was used in their 8th to 12th passage after receipt and was cultured in Ham's F12 medium supplemented with 2.5 mM glutamin, 100 U/mL penicillin, 100 μg/mL streptomycin, 100 ng/mL amphothericin B and 10% fetal bovine serum. Cells were grown in 5% CO_2 _in air at 37°C; the medium was changed three times per week.

### Competitive binding assays

Competition assays were performed in 24-well plates using PC3 cells. The cells were cultured until near confluence, and the medium was replaced by 400 μL of reaction medium (RPMI complemented with 2 mg/mL BSA, 4.8 mg/mL HEPES, 1 U/mL penicillin G and 1 μg/mL streptomycin). For the assay, equal volumes of radioactive and non-radioactive ligands were added. The concentration of [^125^I-Tyr^4^]bombesin (74 TBq/mmol; Perkin Elmer Life Science Products, Boston, MA, USA) was 10^-12 ^M. Increasing concentrations (10^-6 ^to 10^-14^) of the GRPR ligand were added. The plates were incubated for 40 min at 37°C with agitation. After the incubation, the reaction medium was removed, and the cells were washed three times with PBS at room temperature. The cells were harvested and counted in a gamma counter (Cobra II auto-gamma counter, Packard, MN, USA). Experiments were realized three times in triplicate. Data were analyzed with GraphPad Prism 5 Software (GraphPad Software, San Diego, CA, USA) to determine the IC_50_. Finally, the K_i _was determined using Cheng and Pursoff's formula [32]. The K_d _value for [^125^I-Tyr^4^]bombesin has been determined from experiments done under similar conditions and is 1.5 × 10^-10 ^M.

### Peptide radiolabeling with ^64^Cu

**Figure 3:**
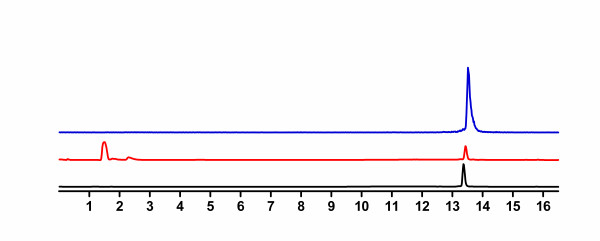
**Representative radio-HPLCs for the purification of ^64^Cu/NOTA-monomer**. Ultraviolet [UV] profile of the starting material (223 nm; absorbance units, black line), UV profile of the purified ^64^Cu/NOTA-monomer (223 nm, absorbance units, blue line), radioactive detection of the purified ^64^Cu/NOTA-monomer (mV, red line).

Our cyclotron facility provides ^64^Cu isotope on a routine basis for research purposes using a target system developed in collaboration with Advanced Cyclotron Systems Inc. (ACSI, Richmond, British Columbia, Canada). ^64^Cu was prepared on an EBCO TR-19 cyclotron (EBCO Technologies, Vancouver, Canada) by the ^64^Ni(p, n)^64^Cu nuclear reaction using an enriched ^64^Ni target electroplated on a rhodium disk [33]. [^64^Cu]CuCl_2 _was recovered from the target following the procedure of McCarthy et al. [34] and converted to [^64^Cu]copper[II] acetate by dissolving the [^64^Cu]CuCl_2 _in ammonium acetate (0.1 M; pH 5.5). Peptides were labeled with ^64^Cu following conditions optimized in our laboratory. Briefly, peptides (5 μg) were dissolved in a 0.1 M ammonium acetate buffer at pH 5.5 with [^64^Cu]Cu(OAc)_2 _(8 to 10 mCi, 296 to 370 MBq) in a total volume of 250 to 300 μL, and then, the resulting solution was incubated at 100°C for 10 min. The labeled product was purified by HPLC using a C-18 column and a radio-detector. The amount of radiolabeled peptide was determined by the peak area of the tracer in the UV-chromatogram compared to the UV peak area of the standard unlabelled peptide (Figure 3). In all cases, starting materials and radiolabeled peptides were separable. The peptide fraction was collected, evaporated and counted in a Capintec radioisotope calibrator (Capintec, Inc., NJ, USA) to calculate the specific activity of the product. Since ^64^Cu-labeled NOTA-dimer 1 and NOTA-dimer 2 were poorly soluble in physiological media, a mixture of DMF-PBS (10/90 *v/v*) was used to solubilize the peptides.

### *In vivo *stability studies

Plasma and *in vivo *stability studies were realized as previously described by our group [35]. Briefly, after peptide reconstitution, studies were carried out by incubating the tracers in mouse plasma for a period of 24 h and by injecting around 15 to 25 MBq (400 to 650 μCi; 100 μL) of ^64^Cu/peptide to female Balb/c mice; 3 mice per peptide. After 24 h, a portion of the incubation mixtures in plasma or blood samples taken from the back paw were quenched with equal amounts of MeCN, chilled (4°C) and centrifuged, and the supernatant was assayed by HPLC. The stability was also determined by radio-TLC directly from plasma and blood samples without further handling; free ^64^Cu and purified radiolabeled peptides were used as standards. The radio-TLCs were eluted on C-18-coated plastic sheets with 0.1 M sodium citrate buffer at pH 5.5 using an instant imager system for the radio-detection.

### Biodistribution studies in Balb/c mice

To determine the *in vivo *GRPR-targeting efficacy of labeled peptides, biodistribution of female Balb/c mice were realized with a minimum of 4 mice for each condition. Briefly, mice were injected with 370 to 740 kBq (10 to 20 μCi; 100 μL) of either ^64^Cu/NOTA-monomer, ^64^Cu/NOTA-dimer 1 or ^64^Cu/NOTA-dimer 2 via the caudal vein. The animals were sacrificed with CO_2 _at 30 min post-injection [p.i.]. Organs of interest were then collected, weighed and measured in a gamma counter. The results were expressed as percentage of the injected dose per gram of tissue [%ID/g].

### Cellular uptake and efflux

Cellular uptake and efflux studies were realized three times in triplicate on PC3 cells in presence of NOTA-monomer and NOTA-dimer 2 radiolabeled with ^64^Cu. First, PC3 cells were seeded in 12-wells plates at a density of 2 × 10^5 ^cells per well 48 h prior to the experiment. Before the experiment, the cells were washed three times with PBS, then 950 μL of culture medium was added. For cellular uptake, PC3 cells were incubated 15, 30, 60, 120 and 240 min with 37 kBq (1 μCi; 50 μL) of radiolabeled peptide per well at 37°C with agitation. Once the incubation was over, the medium was removed, and the cells were washed three times with PBS. The cells were harvested and counted in a gamma counter. The results were expressed as percentage of added dose retained per 10^5 ^cells (%AD/10^5 ^cells). For efflux studies, plated-PC3 cells were incubated 1 h with 37 kBq (1 μCi; 50 μL) of radiolabeled peptide. Then, the cells were washed with PBS and fresh medium was added. After 0, 15, 30, 60, 120 and 240 min, the cells were washed thrice with PBS. Finally, the cells were harvested and counted in a gamma counter. The results were expressed as percentage of activity retained by cells relative to baseline at 0 min.

### PET imaging

PET scans were performed using a LabPET8 (Gamma Medica-IDEAS Inc., Sherbrooke, Quebec, Canada) small-animal scanner with a field of view of 7.5 cm. Female Balb/c nude mice were implanted with 10^7 ^PC3 prostate cancer cells. Cells were injected in 150 μL of Matrigel (BD Biosciences, Mississauga, Ontario, Canada) and PBS (2:1). Tumors were given 3 weeks to grow to the size of 5 mm in diameter. For μPET studies, PC3 xenografted female Balb/c nude mice were injected with 3.7 to 7.4 MBq (100 to 200 μCi; 100 μL) of ^64^Cu/NOTA-monomer or ^64^Cu/NOTA-dimer 2 via the caudal vein under isoflurane anesthesia with a minimum of 3 mice for each tracer. Each animal had a 2-h dynamic scan from the injection. The images were reconstructed by a 2-dimensional MLEM algorithm implemented on an analytically derived system matrix [36]. Region of interest [ROI] was traced for tumor, liver, kidney and muscle. The activity contained in each organ was measured at multiple time points, resulting in time-activity curves.

### Biodistribution studies in PC3 tumor-bearing Balb/c nude mice

Tumor-bearing Balb/c nude mice were injected with 370 to 740 kBq (10 to 20 μCi; 100 μL) of ^64^Cu/NOTA-monomer and ^64^Cu/NOTA-dimer 2 via the caudal vein and sacrificed with CO_2 _at different periods of time after injection. Organs of interest were then collected and weighed. -Radioactivity was measured in a gamma counter. The blocking experiments were realized by co-injecting 0.1 μmol of non-radiolabeled peptide. The results were expressed as %ID/g with a minimum of 3 mice for each condition.

## Results

### Peptide synthesis

NOTA-monomer, NOTA-dimers 1 and 2 were prepared with overall yields of 38, 28 and 31%, respectively, based on the substitution rate of the resin determined photometrically from the amount of Fmoc chromophore released upon treatment of the resin with piperidine/DMF. According to analytical HPLC, the purity was 99% for all peptides as reported in Table 1. The purity of the crude south BBN peptide segment was 84%. The peptide was used for the coupling without further purification; the partially protected peptide degrades when purified by HPLC. All measured peptide masses are in agreement with the calculated mass values (Table 1).

**Table 1:** Analytical data for NOTA-BBN(6-14) monomer and dimmers

Peptide	**[M]**^**+**^		Yield	**Purity**^b^	**K_i_**^c^	Labeling
	**Calcd**	**Found^a^**	**(%)**	**(%)**	**(nM)**	**yield**^d ^**(%)**

Bombesin					0.59 ± 0.32	

NOTA-monomer	1,570	1,571	38	99	2.51 ± 1.54^e^	> 95

South BBN peptide segment	1,879	1,880		84^f^		

NOTA-dimer 1	2,976	2,976	28	99	2.00 ± 1.59	> 95

NOTA-dimer 2	3,122	3,122	31	99	1.76 ± 1.30	> 95

^a^Mass values were obtained by MALDI TOF mass spectroscopy or LC/MS/MS. ^b^Purity was determined by HPLC analysis. ^c^Affinities for GRPR were determined with [^125^I-Tyr^4^]bombesin in PC3 human prostate cancer cell line. ^d^Labeling yield (not decay-corrected) was determined by radio-HPLC analysis based on ^64^Cu starting activity. ^e^Similar K_i _values were obtained for the free chelate (1.30 ± 0.74) and the 'cold' Cu/NOTA-monomer (1.60 ± 0.59) when tested in triplicate in human breast cancer T47D cells. ^f^HPLC yield of the crude peptide. NOTA, 1,4,7-triazacyclononane-1,4,7-triacetic acid; K_i_, inhibition constant.

### Competitive binding assays

All three peptide conjugates inhibited the binding of [^125^I-Tyr^4^]bombesin to GRPR of PC3 cells in a concentration-dependant manner. The K_i _values for NOTA-monomer, NOTA-dimer 1 and NOTA-dimer 2 were 2.51 ± 1.54, 2.00 ± 1.59 and 1.76 ± 1.30 nM, respectively (see Table 1). Natural bombesin was used as a standard, and a K_i _value of 0.59 ± 0.32 nM was obtained under the same conditions (Table 1). No significant difference was observed between the different compounds in terms of GRPR affinity.

### Peptide radiolabeling with ^64^Cu, purification and *in vivo *stability

**Figure 4:**
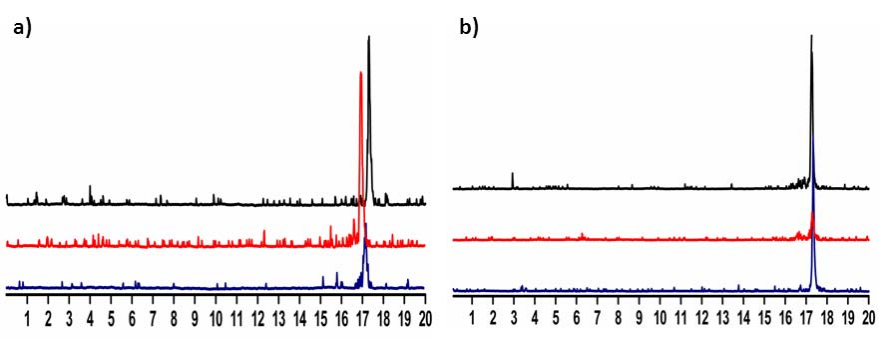
**Representative HPLC radiometric profiles of stability studies**. (**a**) ^64^Cu/NOTA-monomer and (**b**) ^64^Cu/NOTA-dimer 2 after final formulation (black line), after incubation in mouse plasma (24 h, red line) and after 1 h *in vivo *(blue line).

**Figure 5:**
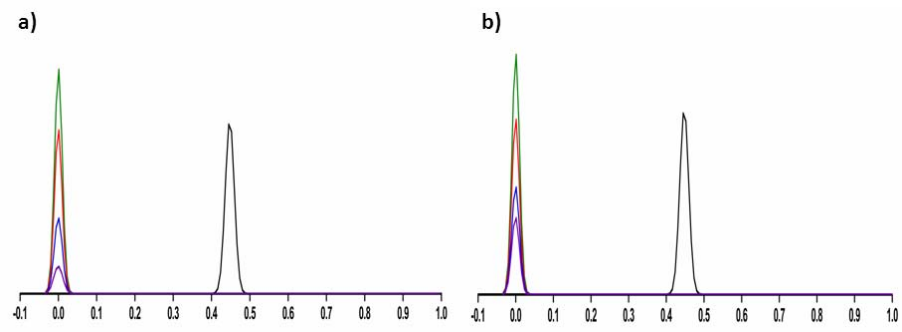
**Representative radio-TLC of stability studies**. (**a**) ^64^Cu/NOTA-monomer and (**b**) ^64^Cu/NOTA-dimer 2 after final formulation (green line), 24 h incubation in mouse plasma (red line), 1 h *in vivo *(blue line), 24 h *in vivo *(purple line) and free ^64^Cu (black line).

All NOTA-peptides were successfully radiolabeled with ^64^Cu with yields not decay corrected greater than 95% (Table 1). The specific activities measured were 74 to 93 TBq/mmol (2,000 to 2,500 Ci/mmol) for NOTA-monomer, and 93 to 130 TBq/mmol (2,500 to 3,500 Ci/mmol) for ^64^Cu/NOTA-dimers 1 and 2. Figure 4 shows radio-HPLC chromatograms of ^64^Cu/NOTA-monomer and ^64^Cu/NOTA-dimer 2. The two tracers were stable in mouse plasma over 24 h and *in vivo *over 1 h (Figure 4a, b). The amount of radiolabeled peptide in mouse blood was not sufficient after 24 h to run a radio-HPLC. Instead, stability results were performed by radio-TLC using free ^64^Cu and purified radiolabeled peptides as standards. Figure 5 shows radio-TLC chromatograms of ^64^Cu/NOTA-monomer and ^64^Cu/NOTA-dimer 2 at various time points in mouse plasma and *in vivo*. The absence of free ^64^Cu *in vivo *24 h p.i. confirmed that ^64^Cu/NOTA complexes of the monomer and the dimer 2 are stable (Figure 5). No metabolites were found under all conditions when the stability was followed by radio-TLC.

### Biodistribution in Balb/c mice

**Figure 6:**
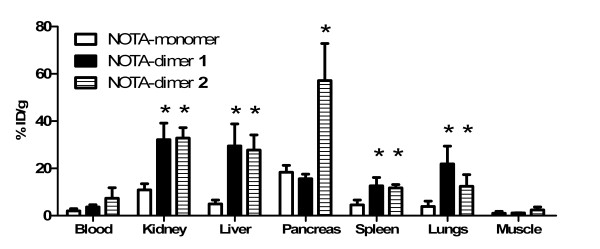
**Biodistributions of ^64^Cu-labeled NOTA-monomer, NOTA-dimer 1 and NOTA-dimer 2**. Biodistributions are at 0.5 h post-injection in Balb/c female mice (four mice/group). Results are presented as mean %ID/g ± SD. The p value refers to the difference between NOTA-dimer 1 (black filled square) and NOTA-monomer (empty square) or NOTA-dimer 2 (stripped filled square) and NOTA-monomer. Asterisk, *p *< 0.05.

The GRPR-targeting *in vivo *efficacy of ^64^Cu-labeled peptides was first tested by biodistribution in female Balb/c mice 30 min p.i. using the pancreas, a GRPR-rich tissue, as a target for specific receptor-mediated accumulation. We also determined the biodistribution profiles of our peptides (Figure 6). Both dimers present higher liver, spleen, lung and kidney uptake. Pancreas uptake were respectively 18.4 ± 2.9, 15.6 ± 2.0 and 57 ± 16%ID/g for NOTA-monomer, NOTA-dimer 1 and NOTA-dimer 2. NOTA-dimer 2 exhibits a 3.6-fold higher pancreas uptake than NOTA-monomer and NOTA-dimer 1.

### Cellular uptake and efflux

**Figure 7:**
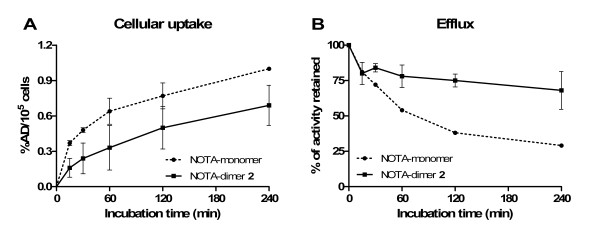
**Cellular uptake (A) and efflux (B) of ^64^Cu-labeled NOTA-monomer and NOTA-dimer 2**. NOTA-monomer, filled circle; NOTA-dimer 2, filled square. Cellular uptake and efflux on PC3 cells (*n *= 3).

To further investigate the polymeric effect observed, we studied cellular uptake and efflux of NOTA-monomer and NOTA-dimer 2 on PC3 cells. The expected advantages of multimeric compound are a higher uptake and retention of the peptide on GRPR-expressing tumor cells. Results are presented in Figure 7. For uptake studies, a significantly higher cellular uptake is observed for the labeled NOTA-monomer at multiple time points (*p *< 0.05). However, efflux studies demonstrate a higher retention of the labeled NOTA-dimer 2 when compared to NOTA-monomer at 1, 2 and 4 h (*p *< 0.05).

### PET imaging

**Figure 8:**
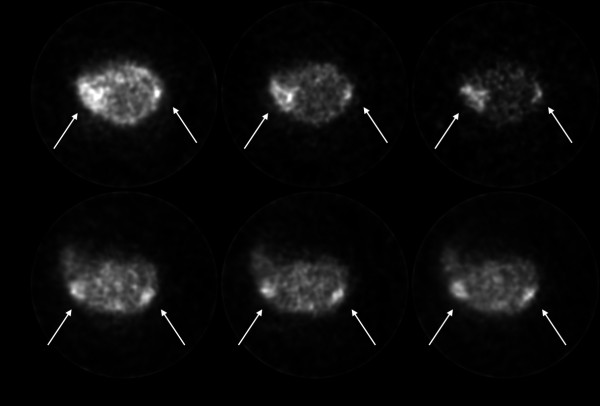
**Decay-corrected transaxial micro-PET images**. Images of PC3 tumor-bearing mice at 30, 60 and 120 minutes post-injection of ^64^Cu/NOTA-monomer or ^64^Cu/NOTA-dimer 2.

**Figure 9:**
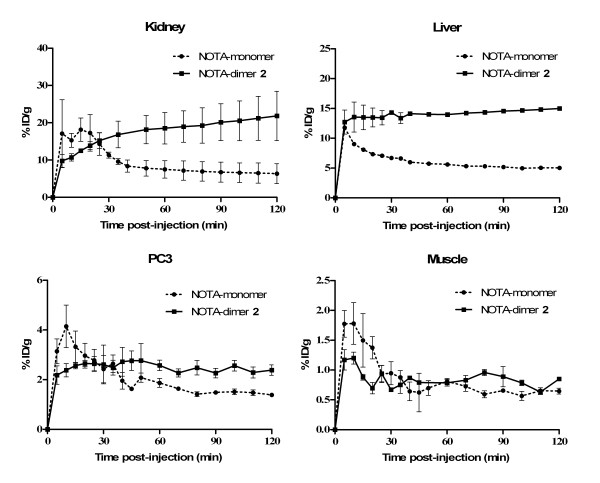
**PET-derived time-activity curves**. Liver, kidney, muscle and PC3 tumor of tumor-bearing mice injected with ^64^Cu/NOTA-monomer (filled circle) or ^64^Cu/NOTA-dimer 2 (filled square).

Representative decay-corrected transaxial images at 30, 60 and 120 min after injection are shown in Figure 8. White arrows indicate the location of the PC3 tumors which were clearly visible at all times with both radiolabeled tracers. From these images, it is evident that the monomer is eliminated from non-target tissue faster than the dimer. Figure 9 presents time-activity curves of liver, kidney, muscle and PC3 tumor with both tracers for the 2-h dynamic scan. From these results, no significant difference was observed between both tracers in terms of muscle accumulation. However, ^64^Cu/NOTA-dimer 2 exhibits higher liver (*p *< 0.05) and kidney (*p *< 0.05) uptake than the ^64^Cu/NOTA-monomer. PC3 tumor uptake profiles are slightly different for both tracers during the first hour p.i.; the monomer exhibits higher uptake during the first half-hour that decreases rapidly to be lower for the next 30 min when compared to NOTA-dimer 2. After 1 h, the dimer exhibited higher retention at the tumor site, in accordance with the higher retention of the NOTA-dimer 2 in cell efflux studies (Figure 7).

### Biodistribution in Balb/c nude mice

In order to validate the results obtained by PET imaging, biodistribution in PC3 tumor-bearing female Balb/c nude mice was realized for ^64^Cu/NOTA-monomer at 0.5 h and ^64^Cu/NOTA-dimer 2 at 0.5 h and 2 h. Results are presented in Table 2. ^64^Cu/NOTA-monomer displayed fast blood clearance with 1.35 ± 0.47%ID/g remaining in the blood at 0.5 h after injection. Blocking studies revealed an increased uptake of ^64^Cu/NOTA-monomer in all organs except pancreas. Ratios of tumor-to-blood and the tumor-to-muscle between unblocked and blocked mice decreased for this tracer. The uptake in the blood, kidney, liver, spleen, lungs and tumor is higher for ^64^Cu/NOTA-dimer 2. A modest decreased uptake was observed at the PC3 tumor site for the dimer 2 with co-injection of 0.1 μmol of non-radiolabeled peptide, but the tumor-to-blood ratio between unblocked and blocked mice significantly diminished. The uptake in the pancreas, which is known to express GRPR, was high and specific for the dimer 2. Surprisingly, a significant reduced uptake was also observed in the liver.

**Table 2:** Biodistribution and tumor to non-target organ ratios for ^64^Cu/NOTA-monomer and ^64^Cu/NOTA-dimer 2

Organ	^64^Cu/NOTA-monomer		^64^Cu/NOTA-dimer 2		
	
	30 min		30 min	120 min	
	
	Unblocked	**Blocked**^a^	Unblocked	Unblocked	**Blocked**^a^
Blood	1.35 ± 0.47	12.72 ± 3.42	3.13 ± 0.67	1.34 ± 0.15	3.32 ± 2.78

Plasma	2.48 ± 0.63	21.69 ± 6.16	5.27 ± 1.27	2.45 ± 0.27	6.14 ± 4.89

Adrenal	4.29 ± 1.13	16.57 ± 0.37	9.23 ± 5.01	11.56 ± 4.91	3.63 ± 2.43

Fat	0.65 ± 0.54	3.22 ± 4.29	1.86 ± 0.81	0.68 ± 0.61	3.46 ± 2.74

Kidney	10.42 ± 1.29	75.13 ± 36.20	17.02 ± 6.24	26.37 ± 8.13	23.00 ± 5.37

Spleen	1.63 ± 1.82	3.29 ± 0.16	5.31 ± 1.43	5.50 ± 2.61	3.31 ± 0.95

Pancreas	5.10 ± 2.50	4.57 ± 2.65	4.60 ± 0.57	13.35 ± 7.38	1.43 ± 0.61^b^

Liver	5.38 ± 8.12	8.12 ± 0.70	41.79 ± 5.58	23.11 ± 2.36	12.20 ± 2.78^b^

Heart	1.05 ± 0.91	4.65 ± 0.33	4.42 ± 1.59	2.68 ± 0.13	1.58 ± 0.80

Lungs	1.77 ± 1.28	14.26 ± 1.58	7.22 ± 2.73	5.07 ± 1.58	34.42 ± 23.31

Muscle	0.40 ± 0.33	3.45 ± 2.06	1.59 ± 1.16	1.13 ± 0.62	1.41 ± 0.98

Bone	0.43 ± 0.46	1.52 ± 0.58	0.78 ± 0.21	0.83 ± 0.19	1.15 ± 0.44

Brain	0.08 ± 0.07	0.63 ± 0.09	0.38 ± 0.06	0.22 ± 0.05	0.16 ± 0.09

Tumor	1.79 ± 0.46	4.82 ± 0.91	3.95 ± 0.26	6.28 ± 2.87	3.25 ± 1.15

Tumor/blood	1.49 ± 0.41	0.38 ± 0.01^b^	1.39 ± 0.30	4.09 ± 1.79	1.04 ± 0.60^b^

Tumor/muscle	7.42 ± 3.17	1.59 ± 0.44^b^	3.95 ± 1.98	4.46 ± 1.86	2.31 ± 1.46

Tumor/liver	2.07 ± 1.13	0.60 ± 0.13	0.10 ± 0.01	0.22 ± 0.07	0.24 ± 0.09

Tumor/kidney	0.17 ± 0.04	0.07 ± 0.01^b^	0.28 ± 0.09	0.22 ± 0.11	0.12 ± 0.03

Tumor/pancreas	0.44 ± 0.17	1.18 ± 0.32^b^	0.89 ± 0.11	0.36 ± 0.09	1.80 ± 0.39^b^

Biodistribution and ratios are at 30 and 120 min post-injection. ^a^Blocked by injecting 0.1 μmol of non-radiolabeled peptide together with the radiopeptide. ^b^Co-injection significantly lowered the uptake of the same organ for the corresponding tracer (*p *< 0.05).

## Discussion

In this study, we investigated the potential benefits of dimeric BBN-based peptide radio-tracers for GRPR-mediated prostate cancer PET imaging. Multimeric compounds are expected to have higher affinity, when targeting receptor at the surface of tumor cells, and a higher tumor uptake and retention than their monomeric counterparts [26]. The binding affinity for GRPR on PC3 cells of NOTA-monomer, NOTA-dimer 1 and NOTA-dimer 2 was similar in the low nanomolar range. The incorporation of the NOTA chelator seems to have a minimal effect on the receptor binding affinity of the peptides [31]. Competitive binding assays demonstrated no advantage in terms of receptor affinity through dimerization. Previous studies also suggested that homodimers show no or modest improvement in affinity for GRPR [27,28].

The specific activities of the dimers were slightly higher than that of the monomer when calculated on a molar basis. Although the amount of peptide conjugate was kept constant for the labeling, i.e. 5 μg, the quantity of radioactivity varied in each case, explaining the observed variations in specific activity. ^64^Cu/NOTA monomer and ^64^Cu/NOTA dimer 2 are stable after 24-h incubation in mouse plasma and 1 h *in vivo *as no trace of free ^64^Cu or metabolite was detected by radio-HPLC (Figure 4). Although ^64^Cu/NOTA complexes of the monomer and the dimer 2 are stable over 24 h *in vivo *(Figure 5), the stability of the peptide itself cannot be established by radio-TLC because the peptide separation from its potential metabolites may be extremely difficult using this method. The biodistribution in female Balb/c mice demonstrated that our monomeric and dimeric peptides have different biodistribution profiles (Figure 6). Both dimers presented slightly higher blood retention and a significantly increased uptake in the spleen, lungs, kidney and liver as compared to the monomer. These results appear to correlate to a higher molecular weight rather than GRPR-mediated polyvalency effect since none of these organs express high level of GRPR. The same observation was reported by Liu while testing their dimer and tetramer of RGD [37]. Higher uptake in liver for both dimers seems to indicate a different elimination pathway and kinetics. The liver is an important organ in the metabolism of copper. However, *in vivo *stability studies indicated absence of free ^64^Cu in the blood circulation at all the time points studied (Figure 5), demonstrating that the observed liver uptake is due to hepatobiliary excretion of the dimers. Higher uptake in the kidneys for both dimers can be explained by the higher molecular weight since larger molecules are more slowly excreted, but also, under physiological conditions, our dimers are more positively charged than the monomer. Positively charged molecules are known to be more retained in the kidneys than neutral molecule [38] which could also explain our results. Labeled NOTA-dimer 2 exhibited a 3.6-fold higher pancreas uptake compared to NOTA-monomer or NOTA-dimer 1 in Balb/c mice. This augmentation cannot be explained by the molecular weight difference. Therefore, this higher uptake could be associated to polymeric effect. We further investigated this effect on PC3 cells *in vitro *and *in vivo *with NOTA-monomer and NOTA-dimer 2. Since NOTA-dimer 1 did not present any advantage over the two other tracers, we stopped its further development.

During *in vitro *experiments, ^64^Cu/NOTA-dimer 2 exhibited lower cellular uptake but higher tumor retention on PC3 when compared to ^64^Cu/NOTA-monomer. The lower cellular uptake could reflect differences in biological activities between the two peptides. Our efflux experiments supported the multimeric effect: ^64^Cu/NOTA-dimer 2 was retained by PC3 cells longer than ^64^Cu/NOTA-monomer in the same conditions.

PET imaging in PC3 tumor-bearing mice was used to compare the pharmacokinetics and distribution of the monomeric and the dimeric BBN. Dynamic PET was used to obtain time-activity curves describing the activity profile of the tracer for each ROI as a function of time. Time-activity curves for liver, kidney, muscle and tumor with ^64^Cu/NOTA-monomer and ^64^Cu/NOTA-dimer 2 showed that liver and kidney uptake was higher at all times for the dimer, while muscle uptake was similar for both tracers. These results correspond to the pattern observed in the biodistribution studies. Results from cellular uptake studies predicted higher tumor uptake for ^64^Cu/NOTA-monomer. In fact, PC3 tumor uptake was higher for ^64^Cu/NOTA-monomer for the first 30 min post-injection. Afterwards, the tumor uptake of the dimer was significantly higher than the monomer. This can be explained by the slower tumor washout of the dimer. The longer retention of the dimer by PC3 cells observed in the efflux studies is also observed *in vivo*. Blocking studies by biodistribution in PC3 tumor-bearing mice were realized to validate the results obtained by PET imaging, confirming the GRPR-mediated uptake of the tracer. After co-injection of the non-radiolabeled peptide, the radioactivity level of ^64^Cu/NOTA-monomer slightly decreased in the pancreas. Surprisingly, an important increase of ^64^Cu/NOTA-monomer in all other organs was noticed by co-injection of the non-radiolabeled peptide. In this study, 0.1 μmol of non-radiolabeled peptide were co-administered intravenously with radiotracer injection, which apparently led to serious damage to the kidney functions and significant inhibition of blood clearance of the radiotracer, as suggested by a 9-fold increase in the blood radiotracer level and a more than 7-fold increase in the kidney levels over animals not receiving the non-radiolabeled peptide. Therefore, the absence of any blocking effect of the non-radiolabeled peptide in the tumor and other organs may be explained by interference from an increased influx of the radiotracer from the blood. At 1 h after the injection of ^64^Cu/NOTA-monomer, the radiotracer was still in the form of its parent in the blood suggesting that the increased uptake of the tracer may not be related to the uptake of metabolites for the blocking experiments. Meanwhile, lower tumor-to-blood ratio of 0.38 ± 0.01 and tumor-to-muscle ratio of 1.59 ± 0.44 were obtained 0.5 h p.i for blocked ^64^Cu/NOTA-monomer, with 1.49 ± 0.41 and 7.42 ± 0.41 for the unblocking experiments respectively (*p *< 0.05, Table 2), showing that the tumor localization of ^64^Cu/NOTA-monomer was a result of the GRPR. For dimer 2, the uptake in blood, kidney, spleen and muscle is non-specific since no difference is noted by co-injection of the non-radiolabeled peptide. The co-injection of the non-radiolabeled peptide significantly reduced the pancreas uptake from 13.35 ± 7.38 to 1.43 ± 0.61%ID/g (*p *< 0.05, Table 2). Since GRPRs are highly express in this organ, this confirms that pancreas uptake of ^64^Cu/NOTA-dimer 2 is GRPR-mediated. The liver uptake of ^64^Cu/NOTA-dimer 2 was also reduced by co-injection of unlabeled peptide (*p *< 0.05). Since GRPR expression in the liver is very low, it is likely that diminution of liver accumulation may reflect saturation of the hepatobiliary elimination pathway by the non-radiolabeled peptide. PC3 tumor uptake of ^64^Cu/NOTA-dimer 2 was modestly lowered from 6.28 ± 2.87%ID/g to 3.25 ± 1.15%ID/g following the co-injection of the non-labeled peptide, again, indicating a GRPR dependant response.

To demonstrate that the higher tumor retention of ^64^Cu/NOTA-dimer 2 observed *in vivo *is not only due to higher molecular weight, we compared the tumor-to-non-target tissue ratio of radiolabeled NOTA-monomer and NOTA-dimer 2. If tumor uptake was only due to size difference among the monomer and the dimer, then muscle uptake would have increased accordingly, resulting in similar ratios of tumor-to-blood and tumor-to-muscle for both tracers. Results indicate that the ^64^Cu/NOTA-monomer offers the highest tumor-to-muscle ratio during the course of our study (Table 2). ^64^Cu/NOTA-dimer 2 exhibits higher tumor-to-blood ratio after 2 h and is also retained longer at the tumor site. In addition, this tracer displays lower tumor-to-liver ratio and similar ratios of tumor-to-kidney and tumor-to-pancreas than the NOTA-monomer at all time points. These ratios are modulated by the different elimination pathway and kinetics of both tracers.

Multimeric compounds could have enhanced affinity due to statistical rebinding or simultaneous binding to receptors. All peptides in the current research feature only short linkers, limiting the possibility of multiple binding to targets simultaneously. Therefore, statistical rebinding seems to be the major factor explaining the results observed at the pancreas for the dimer 2. The receptor binding of one BBN(6-14) unit will significantly enhance the local concentration of the other BBN(6-14) unit in the vicinity of the receptor which might facilitate further binding. However, statistical rebinding is dependent on the density of receptor present at the surface of the cell. GRPRs are characterized by the presence of high and low affinity states to agonist depending on the coupling of guanine nucleotide (GDP or GTP) on the G-protein [39]. Therefore, available receptors that could bind with our peptides at the tumor are likely even lower than expected. In fact, we observed significantly higher uptake in mouse pancreas, which has a very high density of GRPR, but no difference in uptake for PC3 tumors that have a lower GRPR density [1]. This difference in receptor densities might be a factor reducing the efficacy of our dimer. Longer spacers in the dimer might allow bivalency, implying that both BBN(6-14) units can bind simultaneously to GRPR. However, it is important to note that tumor retention did not seem to be altered by lower receptor densities. It is possible that, in the case of the dimeric tracers, different mechanisms are involved in tumor uptake and retention. Overall, our data show that ^64^Cu/NOTA-dimer 2 presents similar affinity for GRPR and PC3 uptake *in vivo*, greater tumor retention *in vitro *and *in vivo *but also higher uptake in the liver and kidney when compared to ^64^Cu/NOTA-monomer.

## Conclusions

Monomeric and dimeric BBN(6-14) peptides have been successfully synthesized and labeled with ^64^Cu as potential tracers for prostate cancer PET imaging. In this study, we present (to our knowledge, for the first time) *in vivo *characterization of radiolabeled dimeric BBN-based peptides. Both ^64^Cu/NOTA-monomer and ^64^Cu/NOTA-dimer 2 are suitable for detecting GRPR-positive prostate cancer *in vivo *by PET. Tumor retention was improved *in vivo *with ^64^Cu/NOTA-dimer 2 by applying polyvalency effect and/or statistical rebinding. Our study is the first step in developing effective dimeric BBN-based tracers in prostate cancer PET imaging.

## Abbreviations

The abbreviations for the common amino acids are in accordance with the recommendations of [40]. Additional abbreviations: %ID/g: percentage of the injected dose per gram; μPET: micro-positron emission tomography; BBN: bombesin; BBN(6-14): [D-Tyr^6^,βAla^11^: Thi^13^: Nle^14^] bombesin(6-14); BSA: bovine serum albumin; ClHOBT: 6-chloro-1-hydroxy-1H-benzotriazole; DCM: dichloromethane; DIEA: *N,N*-diisopropylethylamine; DMF: *N,N*-dimetylformamide; DOTA: 1,4,7,10-tetraazacyclododecane-*N,N',N'',N'''*-tretraacetic acid; GRP: gastrin-releasing peptide; GRPR: gastrin-releasing peptide receptor; HATU: 2-(1H-7-azabenzotriazol-1-yl)-1,1,3,3-tetramethyluronium hexafluorophosphate; HEPES: 4-(2-hydroxyethyl)-1-piperazineethanesulfonic acid; HPLC: high performance liquid chromatography; *i*-Pr-OH: isopropyl alcohol; K_i_: inhibition constant; MeCN: acetonitrile; NMP: *N*-methyl-2-pyrrolidone; NOTA: 1,4,7-triazacyclononane-1,4,7-triacetic acid; PBS: phosphate-buffered saline; PET: positron emission tomography; PEG: polyethylene glycol; p.i.: post-injection; PyBOP: benzotriazol-1-yl-oxytripyrrolidino phosphonium hexafluorophosphate; ROI: region of interest; TFA: trifluoroacetic acid.

## Competing interests

The authors declare that they have no competing interests.

## Authors' contributions

FP carried out the *in vitro *and *in vivo *experiments, data analysis and drafted the manuscript. DPV carried out the *in vivo *experiments. A-MS carried out peptide synthesis, ^64^Ni-target electroplating and ^64^Cu-labeling. LR participated to the ^64^Ni-target electroplating and ^64^Cu-labeling. BF participated in the conception and design of the study. LeR participated in the coordination of the study and reviewed the manuscript. GB conceived of the study, participated in its design and coordination, and helped in making the draft of the manuscript. All authors have read and approved the final manuscript.
